# Increasing Antenatal Care and HIV Testing among Rural Pregnant Women with Conditional Cash Transfers to Self-Help Groups: An Evaluation Study in Rural Mysore, India

**DOI:** 10.1155/2013/971458

**Published:** 2013-09-01

**Authors:** Purnima Madhivanan, Bhavana NiranjanKumar, Reshma Shaheen, Poornima Jaykrishna, Kavitha Ravi, Savitha Gowda, Vijaya Srinivas, Anjali Arun, Karl Krupp

**Affiliations:** ^1^Department of Epidemiology, Robert Stempel College of Public Health and Social Work, Florida International University, 11200 SW 8 Street, HLS 390W2, Miami, FL 33199, USA; ^2^Public Health Research Institute of India, 89/B, 2nd Cross, 2nd Main, Yadavgiri, Mysore 560021, India; ^3^Department of Health Promotion and Disease Prevention, Robert Stempel College of Public Health and Social Work, 11200 SW 8 Street, HLS 390W2, Miami, FL 33199, USA

## Abstract

*Background*. We describe a one-year evaluation study comparing SCIL intervention of mobile provision of integrated ANC/ HIV testing with an enhanced (SCIL+) intervention of community mobilization strategy providing conditional cash transfers (CCT) to women's SHG for identifying and accompanying pregnant women to mobile clinics. *Methods*. Twenty pairs of villages matched on population, socioeconomic status, access to medical facilities, and distance from Mysore city were divided between SCIL and SCIL+ interventions. The primary study outcome was the proportion of total pregnancies in these villages who received ANC and HIV testing. *Results*. Between April 2011 and March 2012, 552 pregnant women participated in SCIL or SCIL+ interventions. Among women who were pregnant at the time of intervention delivery, 181 of 418 (43.3%) women pregnant at the time of intervention delivery received ANC in the SCIL arm, while 371 of 512 (72.5%) received ANC in the SCIL+ arm (*P* < 0.001); 175 (97%) in the SCIL and 366 (98.6%) in the SCIL+ arm consented to HIV testing (*P* < 0.001). HIV prevalence of 0.6% was detected among SCIL clinic, and 0.9% among attending SCIL+ clinic attendees. *Conclusion*. Provision of CCT to women's microeconomic SHG appears to significantly increase uptake of ANC/HIV testing services in rural Mysore villages.

## 1. Introduction

Almost three decades after the first HIV antibody test, the vast majority of pregnant women in middle and low income countries are still not being tested for HIV as part of their antenatal care [1]. According to the United Nations Population Division, only about 26% of 125 million pregnant women in these countries learn their HIV status prior to delivery [2]. Not only is this knowledge essential to the initiation of prevention of mother-to-child transmission (PMTCT) of HIV services, but also it is an important gateway to antiretroviral therapy for HIV infected mothers and children [3]. Despite widespread scale-up of HIV prevention and PMTCT interventions, uptake of services remains low because a majority of HIV-positive pregnant women continue to be unaware of their status. Unsurprisingly, the group with least access to HIV testing remains rural and poor women who are more vulnerable to infection and less able to access needed services [4].

India is among the top ten countries in the world with the highest burden of pediatric HIV infections [5]. Of 27 million women giving birth each year, only 6 million are tested for HIV [6]. Those with least access to prevention services live in the country's 600,000 rural villages. In a 2011 study, only 9% of rural Indian women had an HIV test during their current pregnancy [7]. Low rates of HIV testing have been found closely associated with a lack of accessible and affordable healthcare [8]. The 2005/2006 National Family Health Survey (NFHS III) found that only 6% of poor rural women received prenatal care and only 13% delivered in a medical institution [9]. Current strategies for increasing HIV testing among rural pregnant women have been largely ineffective [10, 11]. New strategies are needed for identifying and testing pregnant women living in India's more than 600,000 rural villages. 

About 5,000 integrated counseling and testing centers (ICTC) currently provide public HIV testing and PMTCT services in India. Only 12% of these facilities are located in rural areas where 61% of Indians live [12, 13]. A study of Karnataka's health infrastructure illustrates the difficulty: a majority of rural residents were required to travel an average of 40 km to access district-level hospitals where ICTC are typically located [13]. As a result, 63% of rural pregnant women elect private healthcare—a problematic choice because HIV prevention services are almost nonexistent in India's “for-profit” sector [14, 15]. Expectant mothers attending public hospitals also face significant challenges. They are referred to outside laboratories for antenatal investigations and to ICTCs for HIV testing. Complicating things further, they are often required to return up to three days later for results. Finally, if they are found to be HIV infected, they must then visit a government Antiretroviral Therapy Centre before or after being seen by a physician for antenatal care. The challenges are obvious. Structural obstacles including distance, expense, and fear of HIV stigma often discourage many rural pregnant women from ever travelling to a government hospital for antenatal care or HIV testing [16]. Only 48% of indigenous tribal populations, for instance, and 20% of the lowest income Hindu women, attend public facilities where they would have access to HIV prevention services [17]. Each step is a barrier to successful provision of PMTCT and a serious cause for loss-to-followup. Studies from Africa have shown that each stage in an HIV prevention cascade results in a loss of 6–12% of women [18].

The Saving Children and Improving Lives (SCIL) intervention was developed to address many of the current barriers to HIV testing among rural women in Karnataka, India. SCIL delivers integrated antenatal care and HIV testing services directly to rural villages using mobile medical clinics, eliminating expense, reducing loss of wages, and mitigating the difficulty of traveling to a public hospital for ANC and ICTC center for HIV testing. The intervention also mobilizes village support for maternal services and reduces fear of HIV stigma among rural pregnant women. An enhanced version of SCIL (SCIL+) adds an additional feature: conditional cash transfers (CCT) given to local women's microeconomic self-help groups for assisting in mobilizing attendance at the mobile medical clinics.

In the past, use of CCT and women's microeconomic self-help groups in interventions has mainly been confined to development programs [19, 20]. With “traditional” cash assistance, recipients receive benefits because they fall into a particular income range or geographic area. CCT are distributed only if recipients comply with certain requirements. For instance, CCT have been shown to increase attendance at health screenings, nutritional education meetings, and immunization clinics [20, 21]. Studies have shown they can have a positive impact on infant birth weight [21] and nutritional status of the infant [22]. SCIL+ uses CCT in an innovative way. Typically, women's microeconomic self-help groups collect small amounts of money from each member and loan it back on a revolving basis. With the SCIL+ intervention, the entire group earns CCT that can then be loaned to members. By identifying and accompanying pregnant woman to ANC/HIV testing clinics, groups increase their capacity for helping members start new businesses and pay unanticipated expenses. As a strategy, utilizing women's self-help groups is also not new, but here again SCIL+ changes the current paradigm viewing women self-help groups as a distribution network instead of seeing them as a collaboration network that helps implement interventions. This ensures dramatically increased ANC/HIV testing coverage in rural areas.

The paper describes an evaluation study that compares the success of SCIL and SCIL+ interventions for mobilizing pregnant women for ANC and HIV testing in 40 rural Mysore district villages in the south Indian state of Karnataka. 

## 2. Materials and Methods

The primary objective of the evaluation study was to examine the effectiveness of the SCIL+ compared with the basic SCIL intervention for increasing uptake of integrated ANC and HIV testing services among women in rural Mysore district. SCIL+ delivers the same community education and awareness activities followed by mobile clinics offering ANC/HIV testing as SCIL, with an additional community mobilization strategy offering CCT to women's microeconomic self-help groups for identifying and accompanying pregnant women to mobile medical clinics. We hypothesized that the “enhancement” would lead to greater uptake of ANC/HIV testing as compared to the basic SCIL intervention. 

## 3. Ethics Review

This evaluation study was approved by the Institutional Review Board of the Public Health Research Institute of India (Protocol number 2011-03-26-10). All women participating in the study gave written informed consent for ANC/HIV testing. 

## 4. Study Setting

The study was conducted from April 2011 to March 2012 in Mysore district, Karnataka. The district has a population of 2,994,744 persons, of which 1,483,538 are female. About 58.6% of residents live in 1,332 rural villages. Annual per capita income for rural residents is estimated at INR 16,086 (USD $322) and literacy at 63.3%, compared with an all-India annual per capita income of INR 38,005 (USD $760) and literacy rate of 74.0% [23–25]. Rural residents are mainly Hindu (86%), 7% Muslim, and 7% belonging to other religions. The district has a 0.8% HIV prevalence in the general population and 1% prevalence among ANC attendees [26]. A 2009 survey evaluating HIV testing in rural Mysore district found that 89.2% of rural women age 15–49 reported no knowledge of an HIV testing center and 80.3% said that they never had an HIV test [27]. 

## 5. Study Design

**Figure 1:**
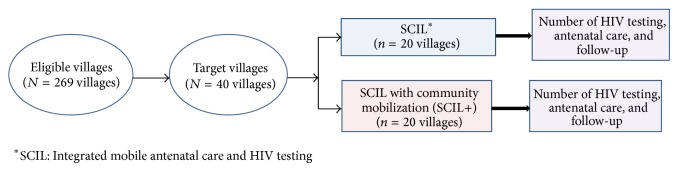
Study design for evaluating the two interventions in rural Mysore, India.

This study employed a quasiexperimental nonequivalent control group design to evaluate whether the SCIL+ was superior to the basic SCIL intervention for increasing uptake of ANC and HIV testing among rural pregnant women. This design is often used when funds are inadequate to conduct a randomized controlled trial but useful data can be gained by comparing different community-level interventions in similar neighboring areas [28]. Both interventions were designed for delivery at the village level, so the study compared interventions in communities closely matched on population size, socioeconomic status, access to public medical facilities, and distance from Mysore city. Forty villages were selected from a sampling frame of 269 villages in Mysore district that met inclusion and exclusion criteria. To be included in the study a village was (a) located more than 10 km outside of Mysore city (to ensure that study villages were rural); (b) having a population size of 1,500–3,000; and (c) not having a public medical facility (Figure 1).

## 6. Participants

The study population included pregnant women, 18 years and older residing in a study village for more than six months. Community education meetings emphasizing the importance of ANC and HIV testing were conducted in all study villages prior to a mobile clinic visit. In SCIL+ villages, study staff met with all women's self-help groups and selected 25 groups in proximity to locations where mobile medical clinics would operate, with a minimum of one group in each village. On the day of the mobile medical clinic, pregnant women who came to access services were screened for eligibility. All eligible women were informed about the study and, if interested, underwent an informed consent process in a private location. 

## 7. Intervention Description

### 7.1. SCIL Intervention

The following activities comprising the SCIL intervention were carried out two times in twenty selected villages during the one-year study.

#### 7.1.1. Community Education and Awareness

 Meetings that included street theatre, presentations on antenatal care and HIV, and participatory activities were conducted in study villages one day prior to arrival of mobile ANC/HIV testing clinics. They were conducted in the local language of *Kannada* and included key messages on birthing preparedness, the importance of antenatal care and HIV testing, recognizing danger signs during pregnancy, and planning ahead for transportation, place of delivery, and healthcare provider. Meetings typically lasted two hours and program staff conducted several times each day.

#### 7.1.2. Integrated Mobile Antenatal Care and HIV Testing Clinics

Intervention staff traveled by minivan with the equipment and supplies necessary for operating a full-service ANC/HIV testing clinic. Pregnant women typically arrived at the clinic site prior to set-up. Staff collected locator information from each pregnant woman to ensure communication of their test results. Women were then provided with group HIV pretest counseling using visual aids designed for low literacy population [29]. Each of the pregnant women underwent an informed consent process in a private setting. Trained interviewers administered a questionnaire to each woman collecting data on knowledge and attitudes about antenatal care, HIV/PMTCT, HIV stigma, institutional delivery, and breastfeeding. Women underwent a detailed physical examination by a physician. A trained nurse phlebotomist collected 2 mL of venous blood for all the antenatal investigations including blood grouping, Rh Typing, random blood sugar, hemoglobin, syphilis, hepatitis B, and HIV testing. Each pregnant woman also provided two mL of urine for prenatal investigations including protein, sugars, and white blood cells. Finally, all women were provided iron, folic acid, and vitamin supplements before they left the clinic. 

#### 7.1.3. Delivery of Laboratory Results and Posttest Counseling

Within 48 hours of a mobile clinic visit, a trained counselor returned back to the same village to deliver results for antenatal and HIV tests to each pregnant woman in person in a private location. The counselor explained the results, helped women cope with any emotional impact of results, and provided appropriate support and referrals. Women found to be HIV-infected were also provided antiretroviral medications for PMTCT and given additional information about HIV-positive women support group and health and social services. Each person referred for additional services was then followed up by phone within a week to find out if she had received needed healthcare and encouraged to do so if she had not done so. 

#### 7.1.4. Followup of Pregnant and Delivered Women

All SCIL clinic attendees were contacted prior to the next clinic visit. Once they delivered, they were followed up in person or by phone within a week and again at three months following delivery. Data were collected on mode of delivery, baby's birth weight, and postnatal healthcare. Information was also collected on breastfeeding practices. 

### 7.2. SCIL+ Intervention

The following activities comprising the SCIL+ intervention were carried out two times in twenty selected villages during the one-year study. The 20 villages in the SCIL+ program received services identical to those in the SCIL intervention along with the following additions.

#### 7.2.1. Social Mobilization Using Women Self-Help Groups

Prior to implementation of the interventions, program staff met with all women's microeconomic self-help groups in SCIL+ program villages to explain the intervention. Groups that expressed interest and capacity to participate (i.e., having at least 10 active members, regular meetings, and a self-help group bank account) were registered to participate in the program. Two weeks prior and again one day before each mobile medical clinic, women's self-help group leaders were notified and reminded of the upcoming medical clinic. Each time a self-help group member accompanied a pregnant woman to the mobile clinic, they were given vouchers that the group's treasurer could redeem for a cash amount equivalent to USD $2.00. 

## 8. Data Analysis

The primary outcome of the study was to estimate the proportion of pregnant women in the SCIL and SCIL+ interventions who received antenatal care and HIV testing. The denominator for the proportion, the total number of pregnant women in villages during the study period, was obtained from a report provided by the India National Rural Health Mission (NRHM) on all pregnant women registered with the NRHM in these villages from the beginning of April 2011 to the end of March 2012. Data analysis was carried out in Stata 12.0 (Stata Corporation, College Station, TX).

## 9. Results

Between April 1, 2011 and March 31, 2012, 76 community awareness programs were conducted in the villages. Programs were open to all adults, and participation was encouraged among spouses and other family members to increase support for maternal healthcare. Meetings were attended by 1,634 residents (480 men and 1154 women) of SCIL villages and 1,479 occupants (535 men and 944 women) of SCIL+ villages. 

A total of 418 pregnancies were identified in SCIL villages. While 24 of the pregnancies (5.7%) had not been registered with NRHM, 181 (43%) attended one or more SCIL clinics. All women underwent pretest counseling, and 175 (96.7%) agreed to HIV testing and received their results after posttest counseling. Six women refused HIV testing as they had recently been tested for HIV and did not want to be tested again. An HIV prevalence of 0.6% was found among participants in the SCIL study arm. 

In the SCIL+ arm, 512 pregnancies were identified with 133 (26%) not registered with the NRHM. About 371 (72.5%) attended at least one medical clinics. Attendees were accompanied by 197 members of 22 women's self-help groups. Among the pregnant women who attended the medical clinics, 366 (98.6%) agreed on pretest counseling and had their HIV testing done. All of them received their results after posttest counseling. Five women refused to get tested for HIV as they had already been tested.. An HIV prevalence of 0.9% was detected among SCIL+ mobile clinic attendees. In SCIL+ arm, 67% more pregnant women received antenatal care and HIV testing. The reasons for not attending the medical clinics in the two interventions are reported in Table 1. 

## 10. Discussion

Currently almost 80% of pregnant women in India fail to receive an HIV test during their pregnancy [6]. This is a significant public health issue in India because almost 50,000 HIV infected Indian women, mostly living in rural areas, give birth annually without the benefit of interventions to prevent vertical transmission of HIV. Recent studies have shown that as few as 9% of rural women receive HIV testing during their pregnancy [7]. Framed in this way, both SCIL and SCIL+ interventions with 43% and 72% uptake of HIV testing, respectively, show great promise for increasing uptake of life-saving interventions such as PMTCT. 

Further research is needed to explore the efficacy of the SCIL and SCIL+ interventions using a randomized controlled trial. The question of how the cost of mobile delivery of ANC and HIV testing to rural areas compares with the expense of lifetime provision of antiretroviral drugs for HIV-infected infants and the social and medical costs of maternal and infant morbidity and mortality will also require additional study before the interventions are considered more widely. 

This study has the following several weaknesses. (a) Due to financial limitations, SCIL and SCIL+ intervention teams were only able to visit study villages twice during the one-year study. For this reason, one-third or more of women who were pregnant during the period were unlikely to have been able to access the mobile medical clinics. (b) In contrast to a true experimental design, this study lacked random assignment. Without this, internal validity is reduced, and causal claims are difficult to make [30]. (c) We did not compare SCIL or SCIL+ to the current standard of care, so we were unable to infer how interventions increase uptake of ANC and HIV testing compared with services that were currently being offered. (d) A quasi-experimental design is unable to ensure that study arms are equivalent, so intervention arms may have differed in important ways that influenced results. For instance, although we found that the SCIL+ intervention performed better, there is no definitive way of determining if this was because of a superior intervention or whether there were other biases or unmeasured confounding. 

Despite these limitations, the evaluation study has several important strengths. It is the first study we know of that involved CCT to women's microeconomic self-help groups instead of self-help group members. Importantly, the methods studied could also be applied to a wide range of interventions in both public health and the development areas. In addition, quasi-experimental designs like the ones used here are used to explore interventions under real-world situations increasing external validity to study data.

## 11. Conclusion

There is a critical need for novel solutions to increase uptake of ANC and HIV testing among rural pregnant women in India and other low and middle income countries. The SCIL and SCIL+ interventions both show great promise for increasing uptake of ANC and HIV testing and reducing mortality and morbidity among expectant mothers and their infants.

## Figures and Tables

**Table 1:** Reasons for not attending the mobile medical clinics in the two intervention groups in rural Mysore, India.

Reasons	SCIL intervention	SCIL+ intervention
Gone to mother's house for delivery	91	79
Already delivered	8	45
Already received antenatal care	13	7
Went to hospital for antenatal care	1	6
Miscarriage/aborted	2	15
Relocated residence	2	9
Refused to attend	120	1

Total	237	162

## Abbreviations

ANC: Antenatal care
CCT: Conditional cash transfer
HIV: Human immunodeficiency virus
ICTC: Integrated counseling and testing centers
NRHM: National rural health mission
PMTCT: Prevention of mother to child transmission
SCIL: Saving children improving lives
USD: United states dollars.
