# Erratum to “Increasing Antenatal Care and HIV Testing among Rural Pregnant Women with Conditional Cash Transfers to Self-Help Groups: An Evaluation Study in Rural Mysore, India”

**DOI:** 10.1155/2014/964269

**Published:** 2014-02-20

**Authors:** Purnima Madhivanan, Bhavana Niranjankumar, Reshma Shaheen, Poornima Jaykrishna, Kavitha Ravi, Savitha Gowda, Vijaya Srinivas, Anjali Arun, Karl Krupp

**Affiliations:** ^1^Department of Epidemiology, Robert Stempel College of Public Health and Social Work, Florida International University, 11200 SW 8 Street, HLS 390W2, Miami, FL 33199, USA; ^2^Public Health Research Institute of India, 89/B, 2nd Cross, 2nd Main, Yadavagiri, Mysore 560021, India; ^3^Department of Health Promotion and Disease Prevention, Robert Stempel College of Public Health and Social Work, 11200 SW 8 Street, HLS 390W2, Miami, FL 33199, USA

In the abstract of the paper titled “*Increasing antenatal care and HIV testing among rural pregnant women with conditional cash transfers to self-help groups: an evaluation study in rural Mysore, India,*” “*175 (97%) in the SCIL and 366 (98.6%) in the SCIL+ arm consented to HIV testing* (*P* < 0.001)” should be changed to “*175 (41.9%) in the SCIL and 366 (71.5%) in the SCIL+ arm consented to HIV testing* (*P* < 0.001).”

